# Changes in processing speed during early abstinence from alcohol dependence

**DOI:** 10.1177/02698811241254830

**Published:** 2024-05-28

**Authors:** Anna Powell, Harry Sumnall, Cecil Kullu, Lynn Owens, Catharine Montgomery

**Affiliations:** 1 School of Psychology, Liverpool John Moores University, Liverpool, UK; 2 Liverpool Centre for Alcohol Research, Liverpool, UK; 3 Public Health Institute, Liverpool John Moores University, Liverpool, UK; 4 Mersey Care NHS Foundation Trust, Liverpool, UK; 5 University of Liverpool, Liverpool, UK

**Keywords:** Alcohol dependence, processing speed, cognitive function, recovery, abstinence

## Abstract

**Background:**

Processing speed is a task-independent construct underpinning more complex goal-related abilities. Processing speed is impaired in alcohol dependence (AD) and is linked to relapse, as are the functions it underpins. Reliable measurement of processing speed may allow tracking of AD recovery trajectories and identify patients requiring additional support.

**Aims:**

To assess changes in reaction time (RT) from baseline (at the start of a detoxification programme) across early abstinence.

**Methods:**

Vibrotactile RT was assessed in early recovery between days 3 and 7 of treatment in 66 individuals with AD (25 females; aged 19–74, 44.60 ± 10.60 years) and against 35 controls tested on one occasion (19 females; 41.00 ± 13.60), using two multivariate multiple regressions. A mixed multivariate analysis of covariance (MANCOVA) of available AD data (*n* = 45) assessed change in RT between timepoints and between treatment settings (outpatient vs inpatient).

**Results:**

The group (AD vs control) significantly predicted choice RT at baseline and follow-up but did not significantly predict simple RT or RT variability, which is inconsistent with previous findings. At follow-up, mental fatigue was also predicted by the group, and MANCOVA indicated that this had worsened in inpatients but improved in outpatients.

**Conclusions:**

Recovery of RT measures so early in the treatment journey was not in line with previous research which indicates persisting deficits. The interaction between setting and timepoint indicates that despite being typically less medically complex, outpatients require ongoing support and monitoring during their recovery.

## Introduction

Recent reviews identify grey matter reductions in the cortex (particularly the prefrontal cortex (PFC) and anterior cingulate cortex) and insulae in alcohol dependence (AD; Yang et al. (2016), Xiao et al. (2015)). In dependent drinkers, this can lead to cognitive deficits or alcohol-related brain injury (ARBI) and major neurocognitive disorders such as alcohol-related dementia or Wernicke-Korsakoff syndrome (Thompson et al., 2020). ARBI is estimated to affect 35% of those with AD (Wilson et al., 2014). Even in the absence of ARBI, cognitive impairments reduce quality of life (Balthazar et al., 2010) and negatively impact treatment outcomes (Domínguez-Salas et al., 2016).

Research into cognitive function in AD consistently finds impairment of executive function (EF) and processing speed (Crowe et al., 2019; Stavro et al., 2012). EF is an umbrella term for functions that enable individuals to plan and maintain goal-directed behavior (Miyake et al., 2000) and are critical for the maintenance of recovery-directed behaviour (Kravitz et al., 2013). Simple processing speed, for example, which requires manual response to a neutral stimulus, is a task-independent construct that underpins more complex abilities (Fry and Hale, 2000), such as EF, by determining the efficiency of cue interpretation and response selection (Miyake et al., 2000). These functions are all heavily dependent on frontoparietal regions, particularly the PFC (Harris et al., 2008; Silva et al., 2018), in addition to white matter integrity (Magistro et al., 2015) and neural transmission speed in the case of processing speed (Volgushev, 2016).

Impairments in EF reduce an individual’s ability to maintain recovery-directed behaviours (Dawson and Grant, 2000; Pitel et al., 2007) and have been linked to AD treatment outcomes, including relapse and treatment adherence (Rupp et al., 2016). Furthermore, PFC dysfunction has been linked to relapse (Charlet et al., 2013) and overcoming craving (Goldstein and Volkow, 2011). As a construct critical to EF, inefficient processing speed also predicts relapse (Allsop et al., 2000; Durazzo et al., 2008; Tapert et al., 2004). Furthermore, white matter integrity predicted relapse in Zou et al. (2018) and correlated significantly with composite *z*-scores of processing speed in future abstainers, but not relapsers.

Processing speed shows improvement across long-term recovery from AD but is still impaired compared to controls up to and beyond a year (Crowe et al., 2019). However, of these functions, deficits in processing speed and EFs are more likely to characterise the early recovery stage (up to a month) than the middle and later stages, which appear related to verbal and visuospatial processing and memory deficits, and residual deficits in verbal and visual memory, respectively (Crowe et al., 2019). Furthermore, given that 50%–80% of people with AD relapse and that this often occurs during early abstinence, such as in the first few days/weeks following detox (Manning et al., 2016), it is clearly important to examine potentially relevant functions to understand treatment outcomes that occur during this early stage.

Currently, the National Institute for Health and Care Excellence (NICE; UK) clinical guidelines recommend routine cognitive screening of individuals receiving alcohol treatment. However, more formal assessments are only advised if an obvious impairment persists after abstention or reduction in alcohol use (NICE, 2011), despite lower-level impairments being widespread and affecting treatment outcomes. Additionally, the suggested initial assessment tool, the Mini-Mental State Exam, may not be sensitive to frontal lobe dysfunction (Carnero-Pardo, 2014). Suggested alternatives (Heirene et al., 2021), including Montreal Cognitive Assessment (MoCA; Thompson et al. (2020)) or Addenbrooke’s Cognitive Examination (Hsieh et al., 2013), show an educational bias (Carnero-Pardo, 2014).

Processing speed can be assessed via ‘reaction time’ (RT), which can be ‘simple’ (one stimulus, one response) or ‘choice’ (more executive, usually with multiple potential stimuli each requiring a distinct response) (Cepeda et al., 2013). Stimulus and response modality during RT assessments can affect results (Guillot et al., 2010), and noise can be added via a number of technical elements (Holden et al., 2020). RT assessed using vibrotactile stimuli (vibration through touch) reduces competing same-sense distractions (Holden et al., 2020; Tommerdahl et al., 2016), and doing so using dedicated hardware and software (such as Brain Gauge Pro (Cortical Metrics, Chapel Hill, NC, USA, https://www.corticalmetrics.com), used in the current study) gives the most accurate RT with the least variability (Holden et al., 2019). Finally, as the stimuli are not letters, numbers or words, this reduces the risk of educational bias, and while instructions are presented on-screen, the facilitator can additionally explain the task verbally.

Previous research using such a device has highlighted alcohol-related differences and changes in cognitive function, indicating that this technology has potential use in this field (Nguyen et al., 2013; Powell et al., 2021). Indeed, a pilot study by Powell et al. (2021) found that Brain Gauge composite scores most sensitive to early treatment were largely those using RT tasks. Understanding RT change during early recovery and how this compares to the ‘normal’ function (of controls) will contribute to our understanding of treatment outcomes. Treatment setting should also be considered, as those referred for inpatient support are likely to have more severe AD and higher daily alcohol use (and therefore receive a higher dosage of benzodiazepines during treatment), more complex comorbidities, and more episodes of relapse following treatment/abstinence (NICE, 2011), all of which may impair cognitive function further (Duka and Stephens, 2014; Gudayol-Ferré et al., 2022). Therefore, as early recovery is such a vulnerable time, it is important to consider treatment settings at this stage because the groups are still distinct; however, once inpatients leave the facility, differences in treatment received are smaller.

The current study therefore aimed to assess changes in RT from baseline (at the start of a detoxification program) across early abstinence. We hypothesised that (1) compared to 35 controls, AD would perform more poorly (greater scores) on all RT scores at both timepoints and (2) in AD participants, outpatients would demonstrate greater processing speed recovery by T1 than those in the inpatient setting.

## Method

### Design

While a longitudinal design assessed the relationship between processing speed and length of abstinence (*N* = 4 timepoints), lower than expected initial recruitment and high attrition (both largely due to COVID-19) meant that only the first two timepoints had sufficient data, so this became a repeated-measures study. The first timepoints occurred during early treatment, and there was some overlap regarding abstinence length. This was due to various factors, including changing outpatient appointment dates, treatment duration differences, and study postponement resulting from participant illness/availability. Testing occurred (i) T0 at 3.27 ± 1.77 (range = 0–7) days post-admission and (ii) T1 at 7.42 ± 2.69 (3–17) days. Despite the overlap, the two timepoints were significantly different regarding abstinence length when assessed by paired *t*-test [*t*(44) = −13.81, *p* < 0.001]. The final two timepoints occurred between 1–2.5 months, and 3–4 months post-detox; however, these are not included in statistical analyses, due to high attrition.

### Participants

Potential participants with AD were identified by clinicians at either an inpatient or outpatient hospital clinic in Liverpool, UK using convenience sampling. Participants were eligible to take part if they were aged 18+, had an ICD-10 diagnosis of AD, were currently undergoing detoxification from alcohol, and were fluent in English. Exclusion criteria were pregnancy or a condition affecting sensation in their dominant hand. In all, 66 individuals with AD were recruited into the study (25 females; aged 19–74, 44.60 ± 10.60 years; AUDIT total 22–44, 32.90 ± 4.98; SADQ total 18–59, 34.00 ± 11.50). Participants were grouped based on treatment setting (outpatient, *n* = 26 vs inpatient, *n* = 40). See Table 1 for the characteristics of participants. Typically, both treatment pathways involved a medically assisted detox, with Librium (chlordiazepoxide) prescribed to treat withdrawal syndrome, after which patients were offered anti-craving medication. Librium was prescribed to 97.6% (*n* = 40) of inpatients and 96.2% (*n* = 25) of outpatients at T0, and 55.9% (*n* = 19) of inpatients and 81.8% (*n* = 9) of outpatients at T1. There was a high attrition rate due to relapse, unexplained loss of contact, changes to clinical appointment dates, and COVID-19-related issues. Therefore, at T1, there were only 11 outpatients, and 34 inpatients remaining; 4 outpatients, 7 inpatients at T2; and 4 outpatients and 2 inpatients at T3. Data from one inpatient (female, aged 36) were removed from the study due to this person having not completed mood state data at T0, and not having taken part at T1, while another inpatient (male, aged 54) was removed due to not meeting timepoint criteria at baseline testing.

**Table 1.** Characteristics of participants at timepoint 0.

	Controls (*n* = 35)				Outpatient					Inpatient				
Characteristic	Min	Max	*M*	SD	Min	Max	*M*	SD	*n*	Min	Max	*M*	SD	*N*
Age	22	72	41.00	13.60	29	68	45.90	11.20	26	19	74	43.80	10.20	40
AUDIT total*	0	19	6.69	4.72	22	44	29.50	4.14	26	24	40	35.70	3.69	32
SADQ-C total*	0	21	4.56	5.03	18	30	24.70	3.25	26	21	59	42.10	9.77	30
Units per day	—	—	—	—	5	45	22.10	11.30	26	4.5	75	35.10	12.90	40
Age of initiation of problem drinking	—	—	—	—	20	63	38.50	13.00	17	13	72	29.80	12.70	39
Duration of problem drinking (in years)	—	—	—	—	2	37	7.35	8.19	17	0	42	13.90	12.00	39
Age of first drink	—	—	—	—	12	26	15.80	3.48	23	10	18	14.70	2.27	27
Total daily Librium at detox start (mg)	—	—	—	—	40	110	84.60	19.80	26	80	200	145.00	26.40	40
Mood state														
Anxiety	0	17	7.4	4.28	4	21	14.90	4.70	26	5	21	13.40	4.74	40
Depression	0	13	3.69	2.82	0	18	9.65	4.73	26	2	19	10.40	4.26	40
	Count	Percentage	Count		Percentage		*n*		Count		Percentage		*n*	
Gender														
Male	16	45.7	17		65.4		26		24		60.0		40	
Female	19	54.3	9		34.6		26		16		40.0		40	
Substance Use														
None	—	—	13		50.0		26		3		7.5		40	
Smoker	—	—	9		34.6		26		6		15.0		40	
Use of cannabis	—	—	3		11.5		26		3		7.5		40	
User of other illicit substances	—	—	1		3.85		26		28		70.0		40	

* Some individuals in the inpatient setting are missing data for AUDIT total and SADQ total due to inconsistencies in data recorded by the treatment provider.

Control participant data were obtained from a previous study (Powell et al., 2022), which recruited a general population sample. In the current study, individuals from this cohort were age- and gender-matched against AD patients using the SPSS (v 28; IBM Corp (2021)) case–control matching function, by age ± 5 years, which selected 35 controls (19 females; 41.00 ± 13.60).

## Materials

### Demographics

Data were collected on age and gender using questionnaires and patient records.

### Alcohol use

Alcohol use was assessed using total scores on the Alcohol Use Disorders Identification Test (AUDIT; Saunders et al. (1993)), Severity of Alcohol Dependence Questionnaire (SADQ-C; Stockwell et al. (1994)) and total mean daily alcohol use prior to detox initiation (UK standard alcohol units). However, AUDIT and SADQ-C were not available for some patients (see Table 1). Additionally, due to this information being passed on via gatekeepers, it was not possible to calculate Cronbach’s α, as only total scores had been recorded.

### Clinical information

Data on substance use other than alcohol (including cigarette smoking, cannabis use or other illicit substances), and relapse or abstinence, were provided by clinical staff. Additionally, in the instance that a follow-up session was attended, clinical records of current alcohol use status were supplemented via self-report.

### Mood state

The Hospital Anxiety and Depression Scale (HADS; Zigmond and Snaith (1983)) was used to assess state anxiety and depression. In the current study, Cronbach’s α totalled 0.92 at T0 and 0.91 at T1 and ranged from 0.91 to 0.92 at T0 and 0.90 to 0.91 at T1 across items. Regarding Cronbach’s for the subscales, Anxiety scored 0.89 at T0, 0.87 at T1, and Depression was 0.81 at T0, and again at T1. Correlations between state depression (*r* = 0.855, *p* < 0.001) and anxiety (*r* = 0.751, *p* < 0.001) at baseline and follow-up indicated strong positive associations between mood state at T0 and T1.

### Reaction time

RT was assessed using a dedicated vibrotactile perception device the size/shape of a computer mouse (with specialised software and an inbuilt microprocessor) called the Brain Gauge Pro. A test battery designed to recruit the PFC involved the application of vibrotactile stimulation via two cylinders (5 mm in diameter) to the index and middle fingers of the dominant hand. Instructions were given on-screen and also explained verbally, with participants completing a series of practice trials for each task, which upon completion of three correct consecutive answers, would progress to 10 successive trials, separated by a randomised interval or 2–7 s (Kim et al., 2020; Zhang et al., 2011). All participants were able to proceed past the practice trials to the main tasks. Task details are below:

*Simple RT* (at start and end of test battery). Participants pressed the opposite tip (index finger) as soon as they felt a tap on their middle finger (25 Hz, 300 μm, 40 ms; Kim et al. (2020)). This task also generated an *RT variability* score: the standard deviation of 10 trials, indicative of attention (Esterman et al., 2013) and a *mental fatigue* score: comparing whether performance worsened between the first and last simple RT assessment (Pearce et al., 2019).

*Choice RT* Participants pressed the opposite tip as soon as they felt a vibration to either the index or middle finger.

Mean scores (milliseconds) of simple RT, RT variability and choice RT were used in all analyses, as was the composite score of mental fatigue, lower scores in all measures indicate better function.

## Procedure

Potential participants with AD were informed of the study by clinical staff at either an outpatient or inpatient detox setting. If interested, they were introduced to the researcher, who gave more details. After giving informed consent, participants completed vibrotactile tasks and then questionnaires (all of which were administered on a Lenovo V14-IIL 14-inch Laptop, with questionnaires via Qualtrics). AD data collection was conducted over multiple sessions, with the initial testing session occurring near the start of the patients’ detox. Where possible, further testing sessions were conducted on around day 7. For the testing itself, participants who were recruited at the outpatient service were always assessed in a room by the clinic (after their attendance at a routine clinic appointment), whilst those in the inpatient setting were assessed initially at this setting, but with any follow-ups on completion of detox assessed in either residential rehabilitation providers or public libraries. Participants were given a £10 shopping voucher for their time, and the study was approved by both the Health Research Authority (IRAS ID: 274928, R&D ID SP0565) and Liverpool John Moores University Research Ethics Committee (REC ID: 19LJMUSPONSOR0037). Control participants were recruited via the methods described in Powell et al. (2022).

## Statistical analysis

Analyses were conducted using R and RStudio (R version 4.2.2; R Core Team (2022), RStudio version 2022.07.2 + 576; RStudio Team (2022)). Significance was accepted at α ⩽ 0.05. To investigate processing speed recovery compared to control scores, multivariate multiple regressions were conducted for timepoints T0 and T1. Predictors were age, gender, mood state and group (control vs AD), and dependent variables were simple RT, RT variability, choice RT and mental fatigue. Cross-sectional methods were used, as controls in the previously recruited study were tested only once but were compared to AD at both abstinence duration points. For regression assumptions, independence of errors and multicollinearity were met. Violations included homogeneity of variance–covariance matrices, and a minority of plots (37.5%) of residuals displaying possible non-linear relationships and heteroskedasticity, with Q–Q plots indicating somewhat non-normal distributions of residuals. However, regression is often robust to this (Schmidt and Finan, 2018), particularly when the sample size is not small (Pek et al., 2018). Several outliers were identified using studentised residuals. However, none were influential or high leverage when assessed with Cook’s Distance (none >1) or hat values (none ⩾0.2), so all were retained in the analyses.

A mixed 2 × 2 MANCOVA of AD data assessed the difference between T0 and T1, with a group (outpatient vs inpatient) as the between-groups factor, time as the within-groups factor, gender as a covariate, and RT measures as dependent variables. Gender was not included as a factor as this significantly reduced the sample size to below requirements (Tabachnick et al., 2013), so it was included as a covariate (homogeneity of regression was achieved, *p* = 0.29 for the factor × covariate interaction). Homogeneity of regression was not achieved with respect to age (*p* < 0.001) and this was not included as a covariate. Homogeneity of variance was met for each dependent variable, and normality was met for most variables (68.75% of Shapiro–Wilk tests). Violations included homogeneity of variance–covariance matrices, some data correlations indicating multicollinearity (29.2%), and some non-linearity between scatterplots of dependent variables (33.3%), so Pillai’s Trace values are reported. Data and analysis script are available on the LJMU open access data repository [https://doi.org/10.24377/LJMU.d.00000184].

## Results

**Figure 1.**
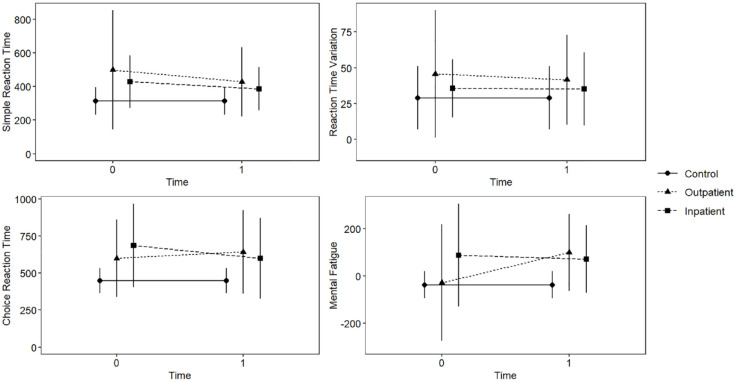
Unadjusted reaction time means (in ms) and standard deviations across groups (controls, outpatients and inpatients), and time. Reaction time (RT) variables were assessed using vibrotactile perception with tactile response. Mental fatigue is a composite measure comparing simple RT at the start and end of the testing session. Timepoints include T0 at around 3 days of treatment and T1 at around 7 days. Controls were tested only once, but their scores are included across each timepoint for comparison. In all cases, higher scores: poorer performance; T0: outpatients (*n* = 26), inpatients (*n* = 40), controls (*n* = 35), T1: outpatients (*n* = 11), inpatients (*n* = 34), controls (*n* = 35).

Figure 1 displays unadjusted RT descriptives for controls, outpatients and inpatients.

### Recovery of RT compared to controls

#### Timepoint 0

A multivariate assessment at T0 (see Table 2) indicated that overall, the group was a significant predictor, while age, gender and mood state were not. Individually, multiple regressions for simple RT, RT variability and choice RT were significant, while mental fatigue was not. Overall explained variance for each of these significantly predicted RT measures was 19.9%, 15.3% and 19.3%, respectively. Individually, the group was a significant predictor of choice RT. Regression coefficients and standard errors are found in Table 2.

**Table 2.** Timepoint 0 multivariate multiple regression predicting RT variables based on age, gender, mood state (HADS anxiety and depression scores) and group (control vs alcohol dependent).

Multivariate analysis of the multiple regressions												
Predictors	Pillai’s *T*	*F*	*df*	*P*	*η_p_* ^2^							
Model												
Constant	0.06	1.60	4, 92	0.18								
Age	0.08	2.09	4, 92	0.09	0.08							
Gender	0.03	0.64	4, 92	0.64	0.03							
Anxiety	0.02	0.55	4, 92	0.70	0.02							
Depression	0.09	2.26	4, 92	0.07	0.09							
Group	0.14	3.61	4, 92	0.01**	0.14							
Individual multiple regressions												
For each Dependent Variable	Unstandardised and standardised coefficients			Squared semi-partial correlation coefficients	Obtained *t* and *p* values		Obtained *R* values			Obtained *F* values		
	*B*	SE *B*	*β*	sr^2^	*t*	*p*	*R* ^2^	*R*	Adj. *R*^2^	*F*	*Df*	*p*
Simple reaction time												
Constant	98.73	96.59			1.02	0.31	0.20	0.45	0.16	4.71	5, 95	<0.001***
Age	4.59	1.81	0.24	0.23	2.53	0.01**						
Gender	−24.33	42.69	−0.05	−0.05	−0.57	0.57						
Anxiety	−1.54	5.81	−0.04	−0.02	−0.27	0.79						
Depression	13.78	6.56	0.31	0.19	2.10	0.04*						
Group	44.87	58.75	0.10	0.07	0.76	0.45						
Reaction time variability												
Constant	1.87	13.19			0.14	0.89	0.15	0.39	0.11	3.42	5, 95	0.01**
Age	0.60	0.25	0.24	0.23	2.42	0.02*						
Gender	−0.45	5.83	−0.01	−0.01	−0.08	0.94						
Anxiety	−0.76	0.79	−0.14	−0.09	−0.96	0.34						
Depression	2.30	0. 90	0.39	0.24	2.57	0.01**						
Group	−1.39	8.02	−0.02	−0.02	−0.17	0.86						
Choice reaction time												
Constant	274.38	108.62			2.53	0.01*	0.19	0.44	0.15	4.53	5, 95	0.001***
Age	4.03	2.04	0.19	0.18	1.98	0.05*						
Gender	20.82	48.01	0.04	0.04	0.43	0.67						
Anxiety	1.27	6.53	0.03	0.02	0.19	0.85						
Depression	−3.17	7.38	−0.06	−0.04	−0.43	0.67						
Group	204.58	66.07	0.40	0.29	3.10	<0.01**						
Fatigue												
Constant	−5.57	90.80			−0.06	0.95	0.09	0.31	0.05	1.95	5, 95	0.09
Age	−1.90	1.70	−0.12	−0.11	−1.12	0.27						
Gender	55.37	40.13	0.14	0.13	1.38	0.17						
Anxiety	6.17	5.46	0.18	0.11	1.13	0.26						
Depression	−7.81	6.17	−0.20	−0.12	−1.27	0.21						
Group	104.68	55.23	0.26	0.19	1.90	0.06						

*N* = 101 (35 controls, 66 AD). RT variables were assessed using vibrotactile perception with tactile response. Mental fatigue is a composite measure comparing simple RT at the start and end of the testing session.

B: unstandardised regression coefficient; SE B: standard error of the coefficient; β: beta coefficient; Adj. *R*^2^: adjusted *R*^2^.

* *p* ⩽ 0.05, **p ⩽ 0.01, ***p ⩽ 0.001.

Effect sizes were calculated for each individual regression, globally for each RT variable model (Cohen, 1988) and also locally for the variable of interest (group), as per Selya et al. (2012). The global model effect size of individual RT variable regressions was medium *f*^2^*s* = 0.25, 0.18, 0.24 when predicting simple RT, RT variability and choice RT, respectively, and small for mental fatigue *f*^2^ = 0.10. The local effect size of the group was small in all models *f*^2^*s* = 0.006, 0.0002, 0.10, 0.04. However, multivariate model effect sizes indicated an overall large effect size for the group, *η*_p_^2^ = 0.14. Due to the impact of the group on choice RT, Pearson’s correlation was used to investigate the relationship between speed and number of correct responses to assess the speed-accuracy trade-off for all three groups (control, outpatient and inpatient). There was a strong negative correlation only for inpatients (*r*(38) = −0.65, *p* < 0.001), but no other group (*ps* > 0.05) indicating that in inpatients at baseline, as speed increased and accuracy decreased.

#### Timepoint 1

A multivariate assessment at T1 (see Table 3) indicated that overall, group and age were significant predictors, while gender and mood state were not. Individually, all four RT variable multiple regressions were significant. Overall explained variance for simple RT, RT variability, choice RT and mental fatigue was 23.4%, 15.0%, 25.3% and 24.7%, respectively. Individually, the group significantly predicted choice RT and mental fatigue, while age predicted all measures. Regression coefficients and standard errors are found in Table 3.

**Table 3.** Timepoint 1 multivariate multiple regression predicting RT variables based on age, gender, mood state (HADS anxiety and depression scores) and group (control vs alcohol dependent).

Multivariate analysis of the multiple regressions												
Predictors	Pillai’s *T*	*F*	*Df*	*p*	*η_p_* ^2^							
Model												
Constant	0.19	4.27	4, 71	<0.01**								
Age	0.16	3.34	4, 71	0.01*	0.16							
Gender	0.01	0.22	4, 71	0.92	0.01							
Anxiety	0.02	0.41	4, 71	0.80	0.02							
Depression	0.05	0.98	4, 71	0.43	0.05							
Group	0.15	3.08	4, 71	0.02*	0.15							
Individual multiple regressions												
For each Dependent Variable	Unstandardised and standardised coefficients			Squared semi-partial correlation coefficients	Obtained *t* and *p* values		Obtained *R* values			Obtained *F* values		
	*B*	SE *B*	*β*	sr^2^	*t*	*p*	*R* ^2^	*R*	Adj. *R*^2^	*F*	*df*	*P*
Simple reaction time												
Constant	183.66	63.21			2.91	<0.01**	0.23	0.48	0.18	4.52	5, 74	0.001***
Age	2.70	1.20	0.25	0.23	2.26	0.03*						
Gender	−5.34	28.88	−0.02	−0.02	−0.19	0.85						
Anxiety	−1.91	4.43	−0.07	−0.04	−0.43	0.67						
Depression	9.66	4.99	0.33	0.20	1.93	0.06						
Group	37.41	32.30	0.14	0.12	1.16	0.25						
Reaction time variability												
Constant	4.35	12.66			0.34	0.73	0.15	0.38	0.09	2.62	5, 74	.03*
Age	0.54	0.24	0.26	0.24	2.25	0.03*						
Gender	3.79	5.78	0.08	0.07	0.66	0.51						
Anxiety	−0.68	0.89	−0.13	−0.08	−0.76	0.45						
Depression	1.51	1.00	0.27	0.16	1.51	0.14						
Group	2.18	6.47	0.04	0.04	0.34	0.74						
Choice reaction time												
Constant	193.56	107.54			1.80	.076*	0.25	0.50	0.20	5.01	5, 74	0.001***
Age	5.94	2.04	.32	.29	2.92	.005**						
Gender	−3.72	49.13	−.01	−.01	−0.08	.94						
Anxiety	−2.52	7.54	−.05	−.03	−0.33	.74						
Depression	8.63	8.49	.17	.10	1.02	.31						
Group	109.44	54.95	.24	.20	1.99	.05*						
Fatigue												
Constant	−161.58	61.75			−2.62	0.011**	0.25	0.50	0.20	4.86	5, 74	0.001***
Age	2.30	1.17	0.22	0.20	1.96	0.054*						
Gender	7.17	28.21	0.03	0.03	0.25	0.80						
Anxiety	4.44	4.33	0.17	0.10	1.03	0.31						
Depression	−1.59	4.88	−0.05	−0.03	−0.33	0.75						
Group	99.48	31.56	0.38	0.32	3.15	<0.01**						

*N* = 80 (35 controls, 45 AD). RT variables were assessed using vibrotactile perception with tactile response. Mental fatigue is a composite measure comparing simple RT at the start and end of the testing session.

B: unstandardised regression coefficient; SE B: standard error of the coefficient; β: beta coefficient; Adj. *R*^2^: adjusted *R*^2^.

* *p* ⩽ 0.05, **p ⩽ 0.01, ***p ⩽ 0.001.

The global model effect size of all individual RT variable regressions was medium, *f*^2^*s* = 0.31, 0.18, 0.34, 0.33, while the local effect size of the group was small in all four models, *f*^2^*s* = 0.02, 0.002, 0.05, 0.13. Again, multivariate effect sizes indicated that the overall effect of the group was large *η*_p_^2^ = 0.15, as was the effect of age *η*_p_^2^ = 0.16. Again, regarding speed-accuracy, there was a strong negative correlation for inpatients (*r*(32) = −0.69, *p* < 0.001), but no other group (*ps* > 0.05) indicating that in inpatients, increased speed was still associated with poorer accuracy.

### Recovery of RT between treatment settings

See Figure 1 to assist with understanding this mixed 2 × 2 MANCOVA. There was a significant interaction effect between time and treatment setting on the combined dependent variables, *F*(4, 40) = 2.55, *p* = 0.05. However, the main effects of time *F*(4, 40) = 1.34, *p* = 0.27, and treatment setting *F*(4, 39) = 1.34, *p* = 0.27 were non-significant, and gender was non-significant as a covariate (*p* = 0.47). Univariate ANCOVAs revealed that there was a significant interaction of medium effect between the two factors on mental fatigue *F*(1, 43) = 5.05, *p* = 0.03, *η*_p_^2^ = 0.11, due to outpatient participants scoring worse (higher) at T1 than at T0, while inpatients improved (scored lower) at this time (see Figure 1). There were no significant interaction effects across the other outcome measures in univariate ANOVAs (*ps* > 0.05).

## Discussion

We investigated the recovery of processing speed in people with AD undergoing outpatient and inpatient-based alcohol treatment. We hypothesised that (1) compared to 35 controls, AD would perform more poorly (greater scores) on all RT scores at both timepoints and (2) in the AD participants, outpatients would demonstrate greater processing speed recovery by T1 than those in the inpatient setting. Hypothesis one was partially supported, as AD performance was poorer than controls at T0 regarding choice RT, but not the other measures, and was poorer in both choice RT and mental fatigue at T1. Hypothesis two was not supported; while there was an interaction between time and treatment setting, this indicated that outpatients performed more poorly on the mental fatigue measure at T1 than baseline, while inpatients had improved.

Impairment in choice RT at baseline is supportive of previous research showing processing speed deficits in AD (Crowe et al., 2019; Stavro et al., 2012), and persisting slower choice RT at T1 in addition to higher mental fatigue is also consistent with expectations. Continued impairment of choice RT indicates that early AD recovery is characterised by impaired performance on RT tasks requiring more executive control (and at least in inpatients, speed came at a cost of reduced accuracy). However, the absence of impairment in simple RT and RT variability compared to controls contrasts with previous research indicating that such impairments persist up to and over a year of recovery (Crowe et al., 2019; Stavro et al., 2012). This is further contrasted by the absence of a difference in recovery of these functions between outpatients and the more clinically complex inpatients from baseline to T1. The reason for this is unclear and perhaps indicates an issue with using vibrotactile stimuli in this context, or a difference between the current cohort, and those studied previously.

Indeed, the range of AUDIT and SADQ-C scores indicates that some control participants were engaging in possibly harmful alcohol use, as the highest AUDIT score was 19, while for SADQ-C it was 21, with scores of eight or above on AUDIT representing hazardous or harmful use (WHO, 2001), and scores between 15 and 30 on the SADQ-C indicating possible moderate physical dependence (NICE, 2011). This may have to some extent reduced the difference in alcohol-related impact on processing speed between the controls and patients assessed in this study, though the group means indicate that overall, alcohol use in the control group was likely to be low risk (non-hazardous on the AUDIT, and none or low dependence on the SADQ-C). Future research could further assess the sensitivity of tasks of varying difficulty to impairment/recovery and compare the utility of different stimuli modalities. Alternatively, the fact that a speed-accuracy trade-off was found only in inpatients indicates a possible inhibitory control deficit (rather than a processing speed deficit) in this group (Kashfi et al., 2017). This may be indicative of pre-existing differences that have a cyclical relationship with the level of AD and alcohol use (López-Caneda et al., 2013) that characterise this group compared to outpatients, which should also be further investigated.

Mental fatigue worsened in AD participants compared to controls by T1. The mixed MANCOVA found that outpatients had worsened by T1, but that inpatients had improved. Mental fatigue is a decrease in cognitive and neural resources due to persistent cognitive demand (Qi et al., 2019), and is experienced as feelings of low energy, or as an increase in effort required to maintain performance (Van Cutsem et al., 2022). The subjective experience can be mitigated by having a break or changing to a less demanding task and is independent of sleepiness (Trejo et al., 2015), which is mitigated by undisturbed sleep (Kumar, 2008). Mental fatigue can reduce well-being (Smith, 2018), physical endurance (Van Cutsem et al., 2017), work performance (McCormick et al., 2012), and leads to changes in motivation, emotion regulation and cognitive function, including EF (Boksem and Tops, 2008; Grillon et al., 2015; Plukaard et al., 2015). Mental fatigue does not always impair performance, as increased effort and individual differences in interest/motivation/personality may moderate its effects (Ackerman and Kanfer, 2009); however, it is still subjectively experienced. The presence of mental fatigue in the absence of simple RT and RT variability deficits indicates that there are alternative measures of function that should be considered. These results also highlight the possibility that current cognitive assessments used in clinical alcohol treatment settings may not capture this, despite being considered when determining treatment setting (NICE, 2011).

Specifically, with regards to EF, mental fatigue is problematic as it reduces an individual’s ability to inhibit a dominant response (Guo et al., 2018), efficiently shift resources between cognitive tasks and plans (van der Linden et al., 2003), replace outdated information in working memory (Pergher et al., 2019) and selectively attend (Faber et al., 2012). This shift in executive control increases the likelihood that decisions will be guided by autonomic ‘bottom-up’ regulatory processes, rather than ‘top-down’ cortical control (van der Linden et al., 2003), a shift that increases relapse risk (Duka and Stephens, 2014). Crucially, mental fatigue has also been associated with an increased risk of alcohol problems (Obeid et al., 2020). Perhaps the expectation for those in outpatient settings to continue their daily lives alongside treatment means they are more likely to cognitively tire during this emotionally and physically draining process. This may, over time, leave the outpatient group at a higher risk for relapse than they should be, considering their fewer complex needs. At least 50% of the outpatients relapsed, which despite their apparent lower need for support, is similar to the 57% relapse rate in the inpatient sample. Additionally, some of those who relapsed from the outpatient group are likely to eventually develop more complex needs and so might require inpatient treatment in the future.

It is also possible that polysubstance use or treatment differences (such as benzodiazepine dose) may explain some of the results. However, this is congruent with the current conclusions, as polysubstance use was higher in inpatients generally, and benzodiazepine dose was more markedly higher in inpatients than outpatients at T1. This would be expected to continue to impair function in inpatients, not improve it, as benzodiazepines have similar pharmacological properties to alcohol (Kreuzer et al., 2019) and link to cognitive deficits (Crowe et al., 2019; Lader, 2014), and polysubstance use is also associated with cognitive deficits in early abstinence (Capella et al., 2015). Alternatively, as alcohol causes cross-tolerance with benzodiazepines (Gravielle, 2016), it is possible that benzodiazepines may not have as much impact on cognitive function in this context in either setting, particularly given that dosage is partly determined by alcohol use, which will also have contributed to patient treatment setting (NICE, 2011). Future research should seek to examine the function between treatment settings and the associated characteristics further, over a longer time period, using validated tasks to assess both recovery and predictability (regarding relapse and other relevant outcomes). The results of such work could give insight into how best to support individuals in each treatment pathway.

There were several limitations to the present study. Recruitment and follow-up numbers were lower than planned and anticipated, which meant that using RT to predict relapse, and within-subjects test of RT change across all timepoints, were not possible. The COVID-19 pandemic resulted in busier outpatient clinics (reducing access to testing rooms) and fewer clinical staff resulting in delayed clinics making recruitment less consistent. Furthermore, post-detox appointments were mostly via telephone as standard practice, rather than face to face, which was not compatible with the study follow-up procedures. We cannot know how RT would be impaired or improved in those individuals lost to follow-up which limits the scope of the study. It is also key to note that the overlap between timepoints for some participants, while significantly different overall, may have confounded the results. However, as the significant MANCOVA interaction includes change over time, the impact of this overlap is less concerning. Additionally, despite the use of Pillai’s Trace to report regression and MANCOVA results, the presence of violations is likely to reduce the statistical power of the models. There were also several possible confounding variables that could not be controlled for, such as time of assessment, as though processing speed may vary due to individual circadian typology (Adan, 1993), appointment time and treatment activities dictated when testing could occur. Another uncontrolled confound was comorbidities/related medication (Marraccini et al., 2016; Nigg et al., 2017). However, controlling these would be difficult, as patients with AD generally have higher rates of comorbid conditions, particularly mental health conditions (Volkow and Li, 2005). Importantly, the dose and frequency of benzodiazepines could not be appropriately controlled for, due to inconsistencies in the level of data provided, and also due to the previously described relationship between dose, alcohol use and treatment setting (NICE, 2011). However, as mentioned, treatment differences in benzodiazepine appear congruent with the current conclusions, and cross-tolerance is likely to reduce the impact of this medication. Finally, controls were only assessed once meaning that the study could not control for potential practice effects in the AD group.

In conclusion, choice RT and mental fatigue showed AD-related deficits during early abstinence. Individuals in this study performed normally on simple RT and RT variability, whilst still experiencing potentially unpleasant and harmful repercussions of cognitive exertion. This is noteworthy, as someone performing normatively on, for example, the MoCA, might be deemed to lack cognitive impairment and thus not need support. That mental fatigue persisted indicates that alternative measures to traditional RT assessments should be considered in this context. Additionally, future research should investigate task difficulty and sensitivity to impairment/recovery, and also the relationship between treatment setting and outcomes, and assess how best to support those with different treatment pathways.
